# Supplementary material for: Why is leptospirosis hard to avoid for the impoverished? Deconstructing leptospirosis transmission risk and the drivers of knowledge, attitudes, and practices in a disadvantaged community in Salvador, Brazil
Source: PLOS Glob Public Health. 2022 Dec 9;2(12):e0000408. doi: 10.1371/journal.pgph.0000408 (PMC10022107; doi:10.1371/journal.pgph.0000408)
Supplement: S1 Checklist — (DOC) [file pgph.0000408.s001.doc]

STROBE Statement—Checklist of items that should be included in reports of ***cross-sectional studies***

|  | Item No | Recommendation |
| --- | --- | --- |
| **Title and abstract** | 1 | (*a*) the word “in a disadvantaged community” in the title shows the study is cross-sectional in design |
|  |  | (*b*) Informative and balanced summary has been provided in the abstract, page 2 (Line 22-39) of the manuscript |
| Introduction | | |
| Background/rationale | 2 | Introduction, Page 4(Line 42-83) section has enough scientific background and rationale why the study is been carried out |
| Objectives | 3 | The later end of the introduction, has the specific objective for the research Page 4 (Line 73-84) |
| Methods | | |
| Study design | 4 | The study design is clearly stated as early as possible, Page 5-6(Line 88-110) |
| Setting | 5 | The study settling and locations has been clearly described in Page 5-6 (Line 88-110) |
| Participants | 6 | Page 5-6 (Line 100-110) clearly describes the sources and method of selection of participants, as well as eligibility criteria |
| Variables | 7 | Page 6-8 (Line 113-158) clearly describes all the variables (response and predictors) in the study. |
| Data sources/ measurement | 8* | *Page 6-8 (Line 113-158*) clearly describe the primary source of KAP data and the secondary source of environmental and epidemiological data |
| Bias | 9 | Information on how we minimized recall bias was provided on Page 5-6 (Line 107-109) |
| Study size | 10 | The method employed was clearly given in Page 5 (Line 100-105) |
| Quantitative variables | 11 | How all quantitative variables were handled was provided in Page 6-8 (Line 121-177) |
| Statistical methods | 12 | (*a*) the relevant information has been provided in Page 7-8 (Line 143-177) |
|  |  | (*b*) the relevant information has been provided in Page 7-8 (Line 143-177) |
|  |  | (*c*) the relevant information has been provided in Page 7-8 (Line 143-177) |
|  |  | (*d*) the relevant information has been provided in Page 7-8 (Line 143-177) |
|  |  | (*e*) N/A |
| Results | | |
| Participants | 13* | (a) the relevant information has been provided in Page 9 (Line 189-193) |
|  |  | (b) relevant information has been provided in Page 9 (Line 191-193) |
|  |  | (c) N/A |
| Descriptive data | 14* | (a) Page 9-10 (Line 189-202) contains reports on demography of study participants |
|  |  | (b) N/A |
| Outcome data | 15* | Outcome events has been reported quantitatively on Page 10-15 (Line 204-279) |
| Main results | 16 | (a) Results has been reported quantitatively on Page 10-15 (Line 204-279) |
|  |  | (*b*) N/A |
|  |  | (*c*) N/A |
| Other analyses | 17 | Results has been reported quantitatively on Page 10-15 (Line 204-279) |
| Discussion | | |
| Key results | 18 | The summary of the key results in reference to study objectives have been discussed on Page 15-19 (Line 281-383) |
| Limitations | 19 | The limitations of the study have been discussed on Page 19 (Line 384-387) |
| Interpretation | 20 | The interpretation of the results has been discussed with the summary Page 10-15 (Line 205-274) |
| Generalisability | 21 | Information on the general validity of the findings have been simultaneously provided on Page 20 (Line 392-401) |
| Other information | | |
| Funding | 22 | Funding details has been sufficiently described on Page 22 (Line 440-443) |

*Give information separately for exposed and unexposed groups.

**Note:** An Explanation and Elaboration article discusses each checklist item and gives methodological background and published examples of transparent reporting. The STROBE checklist is best used in conjunction with this article (freely available on the Web sites of PLoS Medicine at http://www.plosmedicine.org/, Annals of Internal Medicine at http://www.annals.org/, and Epidemiology at http://www.epidem.com/). Information on the STROBE Initiative is available at www.strobe-statement.org.
