# Supplementary material for: Why is leptospirosis hard to avoid for the impoverished? Deconstructing leptospirosis transmission risk and the drivers of knowledge, attitudes, and practices in a disadvantaged community in Salvador, Brazil
Source: PLOS Glob Public Health. 2022 Dec 9;2(12):e0000408. doi: 10.1371/journal.pgph.0000408 (PMC10022107; doi:10.1371/journal.pgph.0000408)
Supplement: S1 Appendix — (DOCX) [file pgph.0000408.s002.docx]

**S1 Appendix File. List of KAP questions**

1. **Sources of information on leptospirosis**

| **Sources of information** |
| --- |
| Community association |
| School |
| Family |
| Health care (ACS/ACE) |
| TV/Radio/Internet |
| Emergency care unit |
| Neighbor |
| Other |

1. **Knowledge questions on leptospirosis**

| **General questions** | |
| --- | --- |
|  | Urine-excreted disease carried by rats |
|  | Disease caused by bacteria |
|  | Transmitted by mosquitoes |
|  | Transmitted person by person |
|  | Infected animals can “pass” to humans |
|  | Disease diagnosed by blood test |
| **Transmission mode** | |
|  | Contact with the urine of infected rats |
|  | Cleaning sewers |
|  | Contact with flood water |
|  | Walking barefoot |
|  | Contact with garbage |
|  | By mosquito bite |
|  | By contact with people who have the disease |
| **Signs and symptoms** | |
|  | Fever |
|  | Body ache |
|  | Headache |
|  | Pain in the legs |
|  | Dehydration |
|  | Nausea and vomiting |
| **Complications** | |
|  | Difficult breathing |
|  | Problem with kidney and liver |
|  | Blisters on the body |
|  | Death |
| **Control and Prevention Practices** | |
|  | Avoid cleaning sewer |
|  | Avoid contact with flood water and trash |
|  | Wear shoes |
|  | Wear rubber boots and gloves |
|  | Eliminate rodents |
|  | Eliminate mosquitoes |
|  | Avoid contact with people who have the disease |

1. **Attitudes questions on leptospirosis**

| **Individual** | |
| --- | --- |
|  | I will use gloves if I have contact with garbage |
|  | I have no worry about walking in the street and having contact with sewage and/or flood water |
|  | I have no worry about wearing rubber gloves and boots while in contact with waste and/or sewage |
|  | I need to use personal protective equipment (rubber boots, gloves, and others) if I have contact with garbage, sewage and /or flood water |
|  | I need to speak with health workers if I suspect I have leptospirosis or know someone who may have this disease |
|  | I don't need to go to the health center if I have a fever during a leptospirosis outbreak in my neighborhood |
|  | I will participate in leptospirosis prevention and control activities offered by the health center |
|  | Leptospirosis has more priority in my life than dengue |
|  | I consider leptospirosis a very serious disease |
|  | I'm afraid of having leptospirosis |
| **Household** | |
|  | I worry if my house is dirty |
|  | I will ensure that my household waste is always closed |
| **Peri-domiciliary** | |
|  | I don't worry if I have rodents outside my household |
|  | I worry if my family participates in cleaning activities outside the household |
|  | I have no worry if the area outside my household is dirty |

1. **Practices questions on leptospirosis**

| **Individual** | |
| --- | --- |
|  | I will not have contact with garbage and /or sewage, flood water unprotected if I have injuries/cuts on my hands/legs |
|  | I eat food if I have contact with garbage without using protection |
|  | I drink water if I have contact with the garbage without using protection |
|  | I wash my hands after having contact with garbage |
|  | I wash the bottle before drinking soda/juice/beer |
|  | I wear gloves and rubber boots if I have contact with garbage, sewage and / or flood water |
| **Household** | |
|  | I keep my food at home in a closed bowl |
|  | I close the cracks (opening) in my home, if there are any |
|  | If there is a mouse hole in my house, people who live in the house will close it |
|  | If I have a rat in my house, I use an illegal poison |
|  | I throw the garbage out |
| **Peri-domiciliary** | |
|  | I cover and / or close the trash bag to avoid the rodents |
|  | I clean the area outside my home to not attract rodents |
|  | I throw garbage in an appropriate place (i.e., container) |
