# Supplementary material for: Why is leptospirosis hard to avoid for the impoverished? Deconstructing leptospirosis transmission risk and the drivers of knowledge, attitudes, and practices in a disadvantaged community in Salvador, Brazil
Source: PLOS Glob Public Health. 2022 Dec 9;2(12):e0000408. doi: 10.1371/journal.pgph.0000408 (PMC10022107; doi:10.1371/journal.pgph.0000408)
Supplement: S1 Table — (DOCX) [file pgph.0000408.s003.docx]

**S1 Table. Summary of knowledge questions on leptospirosis (n= 246)**

| **Knowledge regarding leptospirosis** | **Number** ^a^ | **Percentage (%)**^a^ |
| --- | --- | --- |
| **General questions** |  |  |
| Urine-excreted disease carried by rats | 231 | 93.9 |
| Disease caused by bacteria | 158 | 64.2 |
| Transmitted by mosquitoes | 163 | 66.3 |
| Transmitted person by person | 176 | 71.5 |
| Infected animals can “pass” to humans | 164 | 66.7 |
| Disease diagnosed by blood test | 211 | 85.8 |
| **Transmission mode** |  |  |
| Contact with the urine of infected rats | 237 | 96.3 |
| Cleaning sewers | 221 | 89.8 |
| Contact with flood water | 231 | 93.9 |
| Walking barefoot | 242 | 98.4 |
| Contact with garbage | 235 | 95.5 |
| By mosquito bite | 161 | 65.4 |
| By contact with people who have the disease | 149 | 60.6 |
| **Signs and symptoms** |  |  |
| Fever | 205 | 83.3 |
| Body ache | 212 | 86.2 |
| Headache | 209 | 85.0 |
| Pain in the legs | 193 | 78.5 |
| Dehydration | 150 | 61.0 |
| Nausea and vomiting | 162 | 65.9 |
| **Complications** |  |  |
| Difficult breathing | 116 | 47.2 |
| Problem with kidney and liver | 160 | 65.0 |
| Blisters on the body | 72 | 29.3 |
| Death | 243 | 98.8 |
| **Control and Prevention Practices** |  |  |
| Avoid cleaning sewer | 210 | 85.4 |
| Avoid contact with flood water and trash | 239 | 97.2 |
| Wear shoes | 246 | 100.0 |
| Wear rubber boots and gloves | 242 | 98.4 |
| Eliminate rodents | 239 | 97.2 |
| Eliminate mosquitoes ^b^ | 93 | 37.8 |
| Avoid contact with people who have the disease ^b^ | 114 | 46.3 |
| **Sources of information** |  |  |
| Community association | 3 | 1.2 |
| School | 25 | 10.2 |
| Family | 13 | 5.3 |
| Health care (ACS/ACE) ^c^ | 22 | 8.9 |
| TV/Radio/Internet | 119 | 48.4 |
| Emergency care unit | 1 | 0.4 |
| Neighbor | 59 | 24.0 |
| Other ^d^ | 4 | 1.6 |

^a^ Number (%) of the knowledge about leptospirosis of the participants who answered “Right” for the correct questions and “Wrong” for the incorrect questions.

^b^Number (%) of the knowledge about leptospirosis of the participants who answered “Wrong” to the questions “eliminate mosquitoes” and “avoid contact with people who have the disease”.

^c^ Community Health Workers (ACS) and Endemic Diseases Workers (ACE).

^d^ The category “Other” corresponded to the answers: 2 participants = research project team, 1 participant = work, 1 participant = do not remember.
