# Supplementary material for: Why is leptospirosis hard to avoid for the impoverished? Deconstructing leptospirosis transmission risk and the drivers of knowledge, attitudes, and practices in a disadvantaged community in Salvador, Brazil
Source: PLOS Glob Public Health. 2022 Dec 9;2(12):e0000408. doi: 10.1371/journal.pgph.0000408 (PMC10022107; doi:10.1371/journal.pgph.0000408)
Supplement: S2 Table — (DOCX) [file pgph.0000408.s004.docx]

**S2 Table. Summary of attitudes questions on leptospirosis (n= 248)**

| **Attitudes of participants regarding leptospirosis** | **Number** ^a^ | **Percentage (%)**^a^ |
| --- | --- | --- |
| **Individual related** |  |  |
| I will use gloves if I have contact with garbage | 205 | 82.7 |
| I have no worry about walking in the street and having contact with sewage and/or flood water | 230 | 92.7 |
| I have no worry about wearing rubber gloves and boots while in contact with waste and/or sewage | 208 | 83.9 |
| I need to use personal protective equipment (rubber boots, gloves, and others) if I have contact with garbage, sewage and /or flood water | 231 | 93.1 |
| I need to speak with health workers if I suspect I have leptospirosis or know someone who may have this disease | 236 | 95.2 |
| I don't need to go to the health center if I have a fever during a leptospirosis outbreak in my neighborhood | 220 | 88.7 |
| I will participate in leptospirosis prevention and control activities offered by the health center | 201 | 81.0 |
| Leptospirosis has more priority in my life than dengue^b^ | 179 | 72.2 |
| I consider leptospirosis a very serious disease | 244 | 98.4 |
| I'm afraid of having leptospirosis | 236 | 95.2 |
| **Household related** |  |  |
| I worry if my house is dirty | 245 | 98.8 |
| I will ensure that my household waste is always closed | 232 | 93.5 |
| **Peri-domiciliary related** |  |  |
| I don't worry if I have rodents outside my household | 206 | 83.1 |
| I worry if my family participates in cleaning activities outside the household | 225 | 90.7 |
| I have no worry if the area outside my household is dirty | 209 | 84.3 |

^a^ Number (%) of participants who answered “Yes” to questions about attitudes of towards leptospirosis.

^b^As this is a KAP study for leptospirosis, we will want to know if leptospirosis disease was a priority/important in the lives of the participants.
