# Supplementary material for: Why is leptospirosis hard to avoid for the impoverished? Deconstructing leptospirosis transmission risk and the drivers of knowledge, attitudes, and practices in a disadvantaged community in Salvador, Brazil
Source: PLOS Glob Public Health. 2022 Dec 9;2(12):e0000408. doi: 10.1371/journal.pgph.0000408 (PMC10022107; doi:10.1371/journal.pgph.0000408)
Supplement: S3 Table — (DOCX) [file pgph.0000408.s005.docx]

**Supplementary Materials**

**S3 Table. Summary of practices questions on leptospirosis (n= 248)**

| **Practice regarding leptospirosis** | **Number** ^a^ | **Percentage (%)**^a^ |
| --- | --- | --- |
| **Individual related** |  |  |
| I will not have contact with garbage and /or sewage, flood water unprotected if I have injuries/cuts on my hands/legs | 228 | 91.9 |
| I eat food if I have contact with garbage without using protection | 244 | 98.4 |
| I drink water if I have contact with the garbage without using protection | 235 | 94.8 |
| I wash my hands after having contact with garbage | 242 | 97.6 |
| I wash the bottle before drinking soda/juice/beer | 186 | 75.0 |
| I wear gloves and rubber boots if I have contact with garbage, sewage and / or flood water | 196 | 79.0 |
| **Household related** |  |  |
| I keep my food at home in a closed bowl | 235 | 94.8 |
| I close the cracks (opening) in my home, if there are any | 231 | 93.1 |
| If there is a mouse hole in my house, people who live in the house will close it | 228 | 91.9 |
| If I have a rat in my house, I use an illegal poison | 126 | 50.8 |
| I throw the garbage out | 247 | 99.6 |
| **Peri-domiciliary related** |  |  |
| I cover and / or close the trash bag to avoid the rodents | 238 | 96.0 |
| I clean the area outside my home to not attract rodents | 240 | 96.8 |
| I throw garbage in an appropriate place (i.e., container) | 234 | 94.4 |

^a^Number (%) of participants who answered “Yes” to questions about practices towards leptospirosis.
