# Supplementary material for: Why is leptospirosis hard to avoid for the impoverished? Deconstructing leptospirosis transmission risk and the drivers of knowledge, attitudes, and practices in a disadvantaged community in Salvador, Brazil
Source: PLOS Glob Public Health. 2022 Dec 9;2(12):e0000408. doi: 10.1371/journal.pgph.0000408 (PMC10022107; doi:10.1371/journal.pgph.0000408)
Supplement: Attachment — Submitted filename: Response to Pending Issues_Resolved.docx [file pgph.0000408.s008.docx]

**RESPONSE TO PENDING’ ISSUES**

**Journal Issues Requirements:**

1. Please add a full list of legends for all your Supporting Information files after the references list.

**Response 1:** Thank you for highlight this comment. The full list of legends for Supporting Information files is available after the references list, as below:

**Supporting information**

S1 Appendix File. List of KAP questions

S1 Table. Summary of knowledge questions on leptospirosis (n= 246)

S2 Table. Summary of attitudes questions on leptospirosis (n= 248)

S3 Table. Summary of practices questions on leptospirosis (n= 248)

**Potential Copyright Issues:**

Figure 2: Please confirm (a) that you are the photographer; or (b) provide written permission from the photographer to publish the photo(s) under our CC-BY 4.0 license.

**Response 2:** Thank you for the comment. We can confirm to you that these figures were created by us, using the findings from the analysis of our primary data. We have added a line to clarify this as part of the figure legend. Please see below:

**The authors created these figures using the findings generated from the analysis of their primary data.**

**Figure 1:** please **(a)** provide a direct link to the base layer of the map (i.e., the country or region border shape) and ensure this is also included in the figure legend; and

**Response 3:** Thank you very much for your comment. We have included the full description of base layer map in the figure legend. See below:

**The maps are not copyrighted as they were created by the authors using QGIS 2.18 software. The boundary polygons of Brazil, Salvador and the production of the Salvador income map were downloaded from open and publicly accessible base of IBGE - Instituto Brasileiro de Geografia e Estatistica, on the Geosciences platform which can be accessed at https://www.ibge .gov.br/geociencias/downloads-geociencias.html. The WorldView-3 May 2017 satellite image with 31cm resolution was also used to digitize the study area and sewer line. The image was acquired by the research project / Instituto Gonçalo de Moniz - IGM - Fiocruz Bahia from the company Satmap, with disclosure permitted referencing the Copyrights of DigitalGlobe images.**

**(b)** provide a link to the terms of use / license information for the base layer image or shapefile. We cannot publish proprietary or copyrighted maps (e.g. Google Maps, Mapquest) and the terms of use for your map base layer must be compatible with our CC-BY 4.0 license.

**Response 4:** Thank you very much for your comment. We have included the full description of base layer map in the figure legend. See below:

**The maps are not copyrighted as they were created by the authors using QGIS 2.18 software. The boundary polygons of Brazil, Salvador and the production of the Salvador income map were downloaded from open and publicly accessible base of IBGE - Instituto Brasileiro de Geografia e Estatstica, on the Geosciences platform which can be accessed at https://www.ibge .gov.br/geociencias/downloads-geociencias.html. The WorldView-3 May 2017 satellite image with 31cm resolution was also used to digitize the study area and sewer line. The image was acquired by the research project / Instituto Gonçalo de Moniz - IGM - Fiocruz Bahia from the company Satmap, with disclosure permitted referencing the Copyrights of DigitalGlobe images.**
